# Association of Low Protein-to-Carbohydrate Energy Ratio with Cognitive Impairment in Elderly Type 2 Diabetes Patients

**DOI:** 10.3390/nu16223888

**Published:** 2024-11-14

**Authors:** Antelm Pujol, Pilar Sanchis, María I. Tamayo, Samantha Godoy, Pilar Andrés, Aleksandra Speranskaya, Ana Espino, Ana Estremera, Elena Rigo, Guillermo J. Amengual, Manuel Rodríguez, José Luis Ribes, Isabel Gomila, Félix Grases, Marta González-Freire, Lluís Masmiquel

**Affiliations:** 1 Vascular and Metabolic Diseases Research Group, Endocrinology Department, Son Llàtzer University Hospital, Health Research Institute of the Balearic Islands (IdISBa), 07120 Palma de Mallorca, Spain; antelm.pujol@gmail.com (A.P.);; 2 Laboratory of Renal Lithiasis Research, Department of Chemistry, University of Balearic Islands, Research Institute of Heath Science (IUNICS), Health Research Institute of Balearic Islands (IdISBa), 07120 Palma de Mallorca, Spain; 3 CIBER Fisiopatología de la Obesidad y Nutrición (CIBERObn), Instituto de Salud Carlos III, 28029 Madrid, Spain; 4 Neuropsychology and Cognition, Department of Psychology, Research Institute of Heath Science (IUNICS), University of Balearic Islands, Health Research Institute of Balearic Islands (IdISBa), 07120 Palma de Mallorca, Spain; 5 Neurology Department, Son Llàtzer University Hospital, 07198 Palma de Mallorca, Spain; 6 Neuroradiology Department, Son Llàtzer University Hospital, 07198 Palma de Mallorca, Spainmanuelrodriguez@hsll.es (M.R.); 7 Balearic Research Group on Genetic Cardiopathies, Sudden Death, and TTR Amyloidosis, Health Research Institute of the Balearic Islands (IdISBa), 07120 Palma de Mallorca, Spain; 8 Neuroopthalmology Department, Son Llàtzer University Hospital, 07198 Palma de Mallorca, Spain; 9 Clinical Analysis Department, Son Llàtzer University Hospital, 07198 Palma de Mallorca, Spain; jose.ribes@hsll.es (J.L.R.);; 10 Clinical Toxicology Research Group, Health Research Institute of the Balearic Islands (IdISBa), 07120 Palma de Mallorca, Spain; 11 Translational Research in Aging and Longevity (TRIAL) Group, Health Research Institute of the Balearic Islands (IdISBa), 07120 Palma de Mallorca, Spain; 12 Faculty of Experimental Sciences, Universidad Francisco de Vitoria (UFV), 28223 Madrid, Spain

**Keywords:** type 2 diabetes mellitus, cognitive impairment, diet, protein intake, protein-to-carbohydrate ratio, cognitive performance

## Abstract

Background/Objectives: The relationship between macronutrient intake and cognitive decline in older adults with type 2 diabetes mellitus (T2DM) remains underexplored. Methods: This cross-sectional study aimed to evaluate the association between the protein-to-carbohydrate energy ratio (%E:P) and cognitive impairment among 192 elderly T2DM patients. Cognitive function was assessed using the Montreal Cognitive Assessment (MoCA) and the Self-Administered Gerocognitive Exam (SAGE), while dietary intake data, including (%E:P), was gathered using a validated semi-quantitative food frequency questionnaire. Results: Participants had a mean age of 71 ± 6 years, 46.4% were female, and the median BMI was 30 ± 4 kg/m^2^. After adjusting for confounding variables, patients in the highest (%E:P) tertile showed significantly higher MoCA and SAGE scores compared to those in the lowest tertile (*p* < 0.005). We identified an optimal (%E:P) threshold of 0.375 for predicting cognitive impairment, with a sensitivity of 53% and specificity of 64%. Conclusions: These findings suggest that a lower (%E:P) ratio may be a risk factor for cognitive impairment in elderly T2DM patients. Monitoring this ratio may serve as an early detection tool for cognitive deterioration. Moreover, current protein intake recommendations for older adults with T2DM may be insufficient to prevent cognitive impairment. Further research is needed to establish optimal dietary guidelines for this population.

## 1. Introduction

Addressing the increasing numbers of patients with type 2 diabetes mellitus (T2DM) and cognitive impairment (CI), driven by demographic and lifestyle changes, presents a significant challenge for healthcare systems. The prevalence of T2DM has doubled since the 1980s [1], while dementia impacts 43.8 million people worldwide [2]. This chronic, progressive, and irreversible condition could potentially be mitigated by addressing risk factors early. Targeting factors like lower education, hypertension, hearing loss, smoking, obesity, depression, physical inactivity, diabetes, limited social interaction, excessive alcohol use, traumatic brain injuries, and air pollution could help prevent or delay up to 40% of dementia cases [3].

Diet, as a modifiable lifestyle factor, has shown promise in managing cardiovascular risk factors [4]. Additionally, dietary patterns, such as the Mediterranean-style diet, have been associated with better cognitive function in later life [5]. High caloric intake is linked to an increased cardiovascular disease risk and cognitive impairment, while caloric restriction has been associated with reduced cardiovascular disease and amyloid-β deposition [6,7]. Macronutrients—carbohydrates, fat, and protein—are key determinants of total caloric intake and constitute the largest component of any diet [6]. However, the impact of macronutrient intake relative to total caloric intake on cognitive function in older adults with T2DM has received little attention.

Evidence suggests that a high-protein diet offers health benefits for weight loss and managing cardiometabolic risks, including improved glycemic control in individuals with diabetes, lower blood pressure, and maintenance of metabolic parameters [8]. A low protein intake, however, may elevate the risk of mild cognitive impairment (MCI) or dementia (DE) in elderly individuals [6]. The differential effects of protein and carbohydrate intake on cognitive function may be partly explained by their impact on brain neurotransmitter synthesis, as protein consumption influences plasma levels of tryptophan (Trp) and tyrosine (Tyr), which are precursors to serotonin and catecholamines, neurotransmitters crucial for mood regulation and cognitive processes [9].

Moreover, variations in postprandial glucose response and the glucagon-to-insulin ratio (GIR), which tend to be more stable following protein-rich meals, further support cognitive performance. Research suggests that meals with a balanced or higher protein content may promote more stable glucose metabolism and consistent cognitive function compared to carbohydrate-heavy meals [9,10]. Despite these benefits, long-term observational studies have indicated that high-protein diets may increase the risk of type 2 diabetes, hypertension, and metabolic syndrome [11]. This apparent contradiction could stem from an overemphasis on total protein consumption, without taking into account overall diet quality and total caloric intake [12,13].

Chu et al. observed a positive association between a higher protein-to-carbohydrate ratio and cognitive function in elderly populations in rural China, indicating that sufficient protein intake coupled with moderate carbohydrate intake may support cognitive abilities [14]. Similarly, Yeh et al. found that higher protein intake relative to carbohydrates was linked to reduced odds of subjective cognitive decline (SCD) in a large cohort of U.S. men and women [15]. The evidence consistently shows that a low energy ratio of protein to carbohydrates is associated with cognitive impairment, underscoring the significance of sufficient protein intake and controlled carbohydrate consumption for maintaining cognitive health.

Consequently, the protein-to-carbohydrate energy ratio (%E P:C) has emerged as a key marker of diet quality with significant associations to various health outcomes, including cognitive performance and all-cause mortality [16]. Maintaining an optimal balance between protein and carbohydrates may thus be crucial for not only managing metabolic health but also preserving cognitive function, particularly in individuals with type 2 diabetes. This balance is particularly important for elderly patients, who are at greater risk of cognitive decline and metabolic dysregulation.

This is the first study to examine the association between the %E P:C and cognitive performance in elderly patients with type 2 diabetes. Furthermore, we propose new optimal %E P:C values to prevent cognitive decline in this population. To this end, we analyzed the baseline data from the PHYND study a unicentric, randomized, double-blind, placebo-controlled clinical trial aimed at assessing the effect of oral phytate supplementation on cognitive performance, metabolic control, brain iron deposition, and diabetic retinopathy on elderly patients with mild cognitive impairment and T2DM [17].

## 2. Materials and Methods

### 2.1. Subjects and Design

We performed a prospective, cross-sectional study involving 192 consecutive patients with type 2 diabetes mellitus (T2DM), all aged 60 years or older, recruited from Son Llátzer University Hospital in the Balearic Islands, Spain, between March 2022 and January 2023. Of these, 192 patients were categorized into three groups based on cognitive status: “no cognitive impairment (non-CI)”, “mild cognitive impairment (MCI)”, and “dementia (DE)”. Grouping was determined according to MoCA test cutoff points adjusted for race/ethnicity and years of education [18]. Exclusion criteria included inability to provide informed consent, incomplete anamnestic medical data, recent acute vascular events, and absence of a comprehensive eye exam by an ophthalmologist within the last 6 months.

### 2.2. Clinical, Anthropometric and Laboratory Data

Clinical histories were accessed through electronic medical records. Throughout the study, data including patient anamnesis, laboratory analyses, and physical examinations were collected prospectively. Qualified professionals conducted physical and anthropometric measurements with participants barefoot and wearing light clothing. Morning blood samples were obtained following a 12 h fast, allowed to rest at room temperature for 30 min, and then centrifuged to separate the serum. Hematologic and biochemical analyses were conducted using an automated analyzer (Cell-Dyn Sapphire and Architect ci16200, Abbott, Chicago, IL, USA), while insulin levels were measured via chemiluminescent immunoassay (Advia Centaur, Siemens, Munich, Germany).

Type 2 diabetes was diagnosed in subjects with fasting serum glucose levels of ≥126 mg/dL (7.0 mmol/L), glycated hemoglobin levels of ≥6.5%, or those receiving treatment with hypoglycemic medications [19]. Blood pressure was measured three times after a 5 min rest period with the participant seated quietly, recording the average of the second and third measurements. Participants taking antihypertensive medication or with systolic blood pressure ≥ 140 mmHg and/or diastolic blood pressure ≥ 90 mmHg were classified as hypertensive [20]. Dyslipidemia was defined by the presence of any of the following criteria: LDL cholesterol ≥ 130 mg/dL, HDL cholesterol < 40 mg/dL in men or <50 mg/dL in women, triglycerides ≥ 150 mg/dL, or treatment with lipid-lowering medication. Dyslipidemia was considered in remission if cholesterol and triglyceride levels were below these diagnostic thresholds without lipid-lowering drugs [21]. Chronic kidney disease (CKD) is defined as kidney damage or a glomerular filtration rate (GFR) of less than 60 mL/min/1.73 m^2^ for a duration of 3 months or longer, regardless of the underlying cause [22]. Diabetic retinopathy was diagnosed through a comprehensive ophthalmologic evaluation, which included a dilated slit-lamp examination with biomicroscopy using a handheld lens (90 or 78 diopter), indirect ophthalmoscopy, and additional tests as necessary, such as optical coherence tomography and fluorescein angiography [23].

Dietary intake was assessed using a 137-item, semi-quantitative food frequency questionnaire (FFQ), previously validated in the Spanish population [24]. Foods were grouped into categories such as milk and dairy products, cereals and grain products, vegetables, legumes, eggs, meat and fish, meat products, oils and fats, “fast food”, canned products, fruits, nuts, and beverages. The FFQ asked participants to estimate the frequency of consumption of each food: daily, weekly, monthly, or rarely/never. We recognize that in a sample of elderly individuals with potentially cognitive impairment, data accuracy could be limited due to potential challenges in comprehension or recall of food intake. To mitigate these issues, several strategies were implemented. A healthcare professional reviewed all completed questionnaires to identify potential inconsistencies or missing data. For participants who displayed significant difficulties, the professional provided direct assistance by clarifying questions and helping them accurately recall and record their dietary habits. This approach allowed us to maintain data integrity and minimize the impact of cognitive impairment on dietary data collection.

All completed questionnaires were reviewed by a healthcare professional to verify accuracy and completeness. Medium portions and units (slices, glasses, teaspoons, etc.) were specified for each food.

Nutrient intakes was calculated from the food frequency questionnaires using a food composition table [25] that includes more than 800 foods.

### 2.3. Cognitive Performance Assessment

Cognitive screening was performed using the Montreal Cognitive Assessment Test (MoCA) and the Self-Administered Gerocognitive Exam (SAGE) due to their complementary approaches in detecting cognitive impairment. MoCA is a widely recognized and clinically administered tool, focusing on assessing key cognitive functions such as memory, attention, orientation, and executive functions [26]. This is especially relevant in populations with type 2 diabetes, where deficits in these cognitive areas are common [27]. In contrast, SAGE is a self-administered test that allows participants to complete the assessment independently. This not only facilitates evaluation in contexts where self-administration is preferred or necessary but also helps detect early cognitive issues that may not be as evident in a supervised setting [28]. Additionally, SAGE covers a broader range of cognitive domains, including memory, language, and problem-solving, which complement the areas assessed by MoCA. Together, the combination of MoCA and SAGE enables a more comprehensive evaluation of cognitive impairment in this population by leveraging both the accuracy of a clinician-administered tool (MoCA) and the self-assessment capabilities of SAGE. We suggest that this provides a more holistic perspective on participants’ cognitive status, improving our study’s sensitivity to identifying different levels of cognitive decline. We stratified MoCA results by race/ethnicity and education level before applying a cutoff value for the MoCA score to achieve more accurate cutoffs as suggested by Milani et al. 2018 [18]. SAGE scores of 17 and above are suggestive of a normal condition, scores between 15 and 16 suggested mild cognitive impairment (MCI), and scores of 14 and below was indicative of dementia [28].

### 2.4. Statistical Analysis

The Kolmogorov–Smirnov or Shapiro–Wilk tests, along with normality graphs (histogram, Q-Q plot), were used to determine if the variables followed a normal distribution. Continuous variables are expressed as “mean ± Standard Error” or “median [interquartile range]”. Categorical variables are expressed as “frequency (percentage)”.

For normally distributed variables, the independent samples *t*-test was used to compare variables between two groups. For comparisons between more than two groups, a two-tailed ANOVA test was performed to determine the *p*-value of differences, and the Bonferroni test was applied as a post-hoc test to evaluate differences. For non-normally distributed variables, the Kruskal–Wallis non-parametric test and the Mann–Whitney U test were used. Categorical variables were compared using the chi-square test or Fisher’s exact test to determine differences between groups. *p*-values for trend were calculated using linear regression analysis.

Patients were divided into three groups according to tertiles of protein-to-carbohydrate ratio: low (T1: <0.340), moderate (T2: 0.34–0.415), and high (T3: >0.415). We also stratified patients into three groups based on their MoCA results (normal cognitive function, mild cognitive impairment (MCI), and dementia (DE)), using the MoCA cutoff values suggested by Milani et al. (2018) [18].

Receiver-operating characteristic (ROC) analysis was performed using protein-to-carbohydrate ratio intake as test variable and presence MCI or DE presence as the state variable (normal function as a reference). The optimal cutoff value of protein-to-carbohydrate ratio were determined by the maximum Youden index (J), defined as sensitivity + specificity −1.

Linear regression models were fitted to assess the associations between protein-to-carbohydrate ratio intake and cognitive function, as measured by MOCA and SAGE scales and subscales. For this analysis, tertiles of protein-to-carbohydrate ratio intake were used, considering the first tertile as the reference category. Multivariate models were adjusted for age (years), gender (male/female), duration of diabetes (<5 years/5–10 years/>10 years), education level (<13 years/13–16 years/>16 years), total energy intake (kcal/day).

Binary logistic regression models were used to explore the association of protein-to-carbohydrate ratio intake to MCI or DE, with normal cognitive function as the reference (odds ratio [OR] = 1). Models were adjusted for the same variables as those used in the main analyses. A two-tailed *p*-value less than 0.05 was considered statistically significant. Statistical analyses were performed using SPSS 25.0 (SPSS Inc., Chicago, IL, USA).

## 3. Results

### 3.1. Baseline Patient Clinical Characteristics and Dietary Composition Across MoCA Groups

A total of 192 patients were included in the present study, with a median age of 71 ± 6 years; 46.4% were women, and median BMI was 30 ± 4 kg/cm^2^. The main anthropometric, dietary, and lifestyle data of participants are shown in Table 1. As seen, 88.5% of patients had been living with diabetes for more than 10 years, and 61.5% had less than 13 years of education. Regarding the presence of comorbidities, artherioesclerosis (90.6%), hypertension (85.9%), and chronic kidney disease (19.8%) were the most prevalent ones. Our sample of patients consumed a median of 2006 kcal per day (interquartile range (IQR): 1642–2512), 95 g of protein per day (IQR: 77–114), 89 g of lipids per day (IQR: 70–112), and 246 g of carbohydrates per day (IQR: 205–308). Additionally, the patients reported consuming 621 g of vegetables per day (IQR: 439–792), 529 g of fruit per day (IQR: 472–737), and 101 g of legumes per day (IQR: 43–64). Moreover, Table 1 shows clinical characteristics and dietary composition across MOCA groups. Patients with dementia had a higher age (72; IQR: 71–77; *p*-value for trend 0.01), a lower daily nut consumption (6 g/day; IQR: 0–13; *p*-value for trend 0.03), and a lower amount of protein intake (88 g/day; IQR: 76–102; *p*-value for trend 0.044). They also had a higher percentage of energy consumption from carbohydrates (50%; IQR: 48–54; *p*-value for trend 0.006).

### 3.2. Clinical Characteristics and Cognitive Function Across Tertiles of Protein-to-Carbohydrate Intake

Table 2 describes the clinical characteristics and cognitive function of subjects across tertiles of protein-to-carbohydrate intake. Patients in the highest tertile of intake had higher punctuation of SAGE and MOCA scores than those in the lowest tertiles. Additionally, the percentage of patients with MCI or DE was lower in the highest tertile compared to the lowest tertiles.

### 3.3. Cognitive Function Scales and Subscales Among Protein-to-Carbohydrate Ratio Tertiles

We further explored the association between cognitive function scales and protein-to-carbohydrate ratio (by tertiles and for each increase of 0.2 units) using univariate and multivariate linear regression analysis. Table 3 shows the beta-coefficients and 95% confidence intervals (CI) of the associations between protein-to-carbohydrate intake (by tertiles and for each 0.2-units increase) and MOCA and SAGE values. After adjusting for potential confounders, protein-to-carbohydrate intake was positively and significantly associated with naming, attention, and total punctuation for the MOCA scale and orientation, similarities, executive function, and total punctuation for the SAGE scale.

### 3.4. Optimal Cutoff of Protein-to-Carbohydrate Ratio Associated with MCI or Dementia (vs. Non-CI) in Patients with T2DM

**Figure 1:**
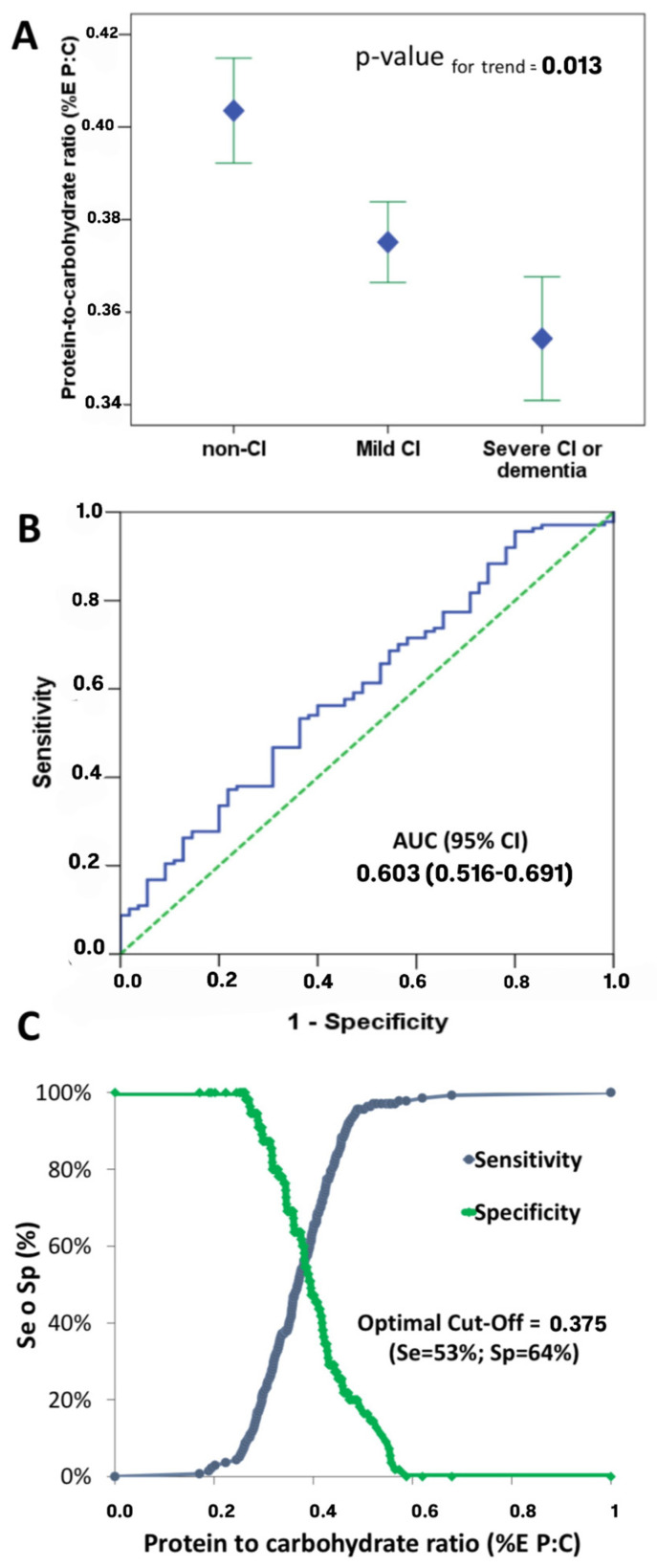
Cognitive function and protein-to-carbohydrate ratio intake. (**A**) Protein-to-carbohydrate ratio intake among cognitive function according MOCA groups. (**B**) Receiver-operating characteristic (ROC) analysis of the of protein-to-carbohydrate ratio (test variable) associated with cognitive impairment or dementia (dependent variable). (**C**) Sensitivity (Se) and specificity (Sp) of the test for different values. The cutoff value that concurrently optimized Se and Sp was % E P:C = 0.375.

In this study, we found that the protein-to-carbohydrate ratio decreases as cognitive function declined (Figure 1A). Patients with dementia had the lowest protein-to-carbohydrate intake compared to those with mild cognitive impairment (MCI) or normal cognitive function. Similarly, individuals with MCI had a lower protein-to-carbohydrate ratio than those with normal cognitive function (Figure 1A).

We used receiver-operating characteristic (ROC) curves (Figure 1B) to determine the optimal cutoff value for protein-to-carbohydrate ratio. MCI or DE served as the state variable. As shown, a %E P:C value lower than 0.375 exhibited a sensitivity of 53% and specificity of 64% (Figure 1C). This indicates that a protein-to-carbohydrate ratio below 0.375 is the optimal threshold associated with the presence of dementia (DE) or mild cognitive impairment (MCI).

### 3.5. Protein-to-Carbohydrate Ratio as a Risk Factor for MCI or Dementia (vs. No-CI) in Patients with T2DM

Binary logistic regression was also performed to examine the association between protein-to-carbohydrate ratio intake and MCI or DE (using normal function as a reference, OR = 1). After adjusting for potential cofounders, protein-to-carbohydrate ratio intake (lower than 0.375 units and per each 0.2 unit decrease) was significantly associated with MCI or DE (Table 4). These results suggest that a lower protein-to-carbohydrate ratio is more strongly associated with an increased likelihood of developing mild cognitive impairment (MCI) or dementia (DE).

## 4. Discussion

Our study is the first to report that a low %E P:C ratio is negatively correlated with MoCA and SAGE scores in elderly subjects with T2DM. Furthermore, we are the first ones to explore the relationship between cognitive domains and %E P:C. We demonstrate that after adjusting for potential confounders, %E P:C was positively and significantly associated with naming, attention, and total score for the MOCA scale and orientation, similarities, executive function, and total score for the SAGE scale. To date, no other studies have assessed the relationship between %E P:C and cognitive domains in elderly subjects with T2DM. The elevated risk of MCI associated with lower protein intake may be linked to mechanisms beyond simple energy supply [29]. Adequate protein consumption may be crucial for maintaining the integrity of neuronal membranes and for neurotransmitter synthesis [29]. For example, tryptophan, which crosses the blood–brain barrier, serves as a precursor for serotonin, a key neurotransmitter in the brain. Studies in mice have shown that the transport of tryptophan across the blood–brain barrier decreases with age [30]. Additionally, inadequate protein intake is associated with declines in modifiable risk factors for dementia, including physical activity [31], sleep [32], stress, depression, and anxiety [33]. Proteins are essential for muscle development, and insufficient protein intake may elevate the risk of frailty and sarcopenia, conditions which are strongly associated with cognitive impairment [34]. Socioeconomic status (SES) often influences protein consumption, with lower SES associated with reduced intake [35]. However, the causal nature of this relationship remains unclear, as there is a lack of rigorous studies exploring the connection between amino acids and cognitive decline. Many proposed mechanisms remain speculative.

The National Health and Nutrition Examination Survey (NHANES) conducted from 2011 to 2014 reported a positive association of dietary protein intake with cognitive function in adults aged 60 years and older [36]. Similar results have been obtained in a healthy elderly population [6,37,38,39]. However, a 24-week clinical trial involving 30 g protein supplementation in elderly individuals showed improvements in reaction time performance in pre-frail and frail elderly individuals but did not lead to improvements in other cognitive functions [40]. Some authors hypostasized that protein may enhance cognitive function by improving glucose homeostasis [6]. Thus, the connection between protein intake and cognitive function is still debated and requires further investigation. T2DM has been associated with a faster decline in cognitive function in older adults, as well as an elevated risk of developing mild cognitive impairment and a higher likelihood of dementia, including Alzheimer’s disease (AD) and vascular dementia [41]. A major gap in the literature is that no observational data and no clinical trials have been published about the relationship between protein intake and cognitive impairment in elderly patients with T2DM.

We propose that a %E P:C value lower than 0.375 (with a sensitivity of 53% and specificity of 64%) is the optimal threshold associated with dementia (DE) or mild cognitive impairment (MCI). While these values indicate that the %E P:C cannot serve as a definitive diagnostic tool, they suggests potential utility as initial risk indicators in this population. These sensitivity and specificity values reflect the inherent limitations of linking macronutrient intake to cognitive status, as cognitive impairment is multifactorial and influenced by additional factors. Therefore, this ratio might be best used in combination with other clinical measures and biomarkers to improve early detection in at-risk individuals. The cutoff of 0.375%E P:C identified as optimal in this research may therefore be most applicable to similar patient profiles, given the unique metabolic and cognitive characteristics associated with T2DM. It is important to note that this threshold might not directly extend to populations without diabetes or those with different metabolic conditions. Future research is encouraged to explore the relevance of this cutoff in diverse demographic groups, including older adults without T2DM or individuals with varying metabolic profiles, to assess the potential need for population-specific dietary guidelines regarding %E P:C ratios.

The association between a 0.2-unit decrease in the protein-to-carbohydrate energy ratio (%E P:C) and an elevated probability of cognitive impairment supports the clinical relevance of dietary monitoring in elderly T2DM patients. Specifically, each 0.2-unit reduction was linked to a significant increase in the odds of MCI or dementia, reinforcing the value of %E P:C as an indicator of cognitive health risk. This relationship suggests that clinicians may consider assessing and optimizing %E P:C ratios as part of routine dietary evaluations, especially in patients at higher risk of cognitive impairment.

The European Society for Clinical Nutrition and Metabolism (ESPEN) advises that aging adults should consume at least 1.0–1.2 g of protein per kilogram of body weight daily [31]. For older adults with acute or chronic illnesses, the recommended intake is increased to 1.2–1.5 g of protein per kilogram of body weight per day, with even higher amounts suggested for those with severe illness or injury [31]. The 2024 American Diabetes Association Standards of Care mentions that there is no evidence to suggest that modifying daily protein intake (usually 1–1.5 g/kg body weight/day or 15–20% of total calories) will lead to health improvements. Research remains inconclusive about the optimal amount of dietary protein for improving glycemic control or reducing cardiovascular disease (CVD) risk [42]. However, these protein amounts are significantly higher than what most older adults typically consume in their daily diets. In fact, most older adults do not meet the Recommended Dietary Allowance (RDA). Approximately 10–25% of older adults consume less protein than the Recommended Dietary Allowance (RDA), while 5–9% consume less than the Estimated Average Requirement (EAR) of 0.66 g/kg per day [43]. It is important to emphasize that the EAR represents the average intake required to meet the needs of a healthy population, meaning that this intake level is inadequate for about half of the population [43]. At present, there are no specific guidelines for protein intake related to cognitive function in patients living with or without T2DM.

We report that patients with DE had a lower daily nut and protein consumption and a higher proportion of energy intake derived from carbohydrates. Several studies show that adults with a low %E P:C ratio were more likely to consume fewer vegetables, leafy greens, beans, dairy products, protein sources, seafood, and plant-based proteins, while having a higher intake of refined grains and added sugars. Their intake of essential nutrients, including calcium, magnesium, potassium, choline, and vitamins A, C, D, and E, was also lower [16]. All those food groups have important nutrients for cognitive function. Moreover, low %E P:C is associated with poor diet quality, substitution of protein food for refined carbohydrates, and increased risk of death from all causes [16].

No differences in caloric intake were reported between cognitive performance subgroups. It is hypothesized that reduced dietary intake in patients with dementia is influenced by factors such as forgetting to eat, losing the ability to use eating utensils, lack of appetite, malabsorption, or poor microbiome quality [44,45,46,47]. Only one study reported that the energy intake of patients with Alzheimer’s disease (AD) dementia was lower than that of controls [48], whereas other studies found no significant differences in energy intake between patients with AD dementia and controls [48,49,50]. Resting energy expenditure (REE), which accounts for approximately 70% of daily energy expenditure, is thought to be higher in patients with AD dementia compared to controls. In fact, Doorduijn et al. (2020) in a cross-sectional study reported that patients with AD dementia and MCI exhibited higher REE compared to controls, despite similar energy intake and physical activity levels [51]. These findings suggest that the negative energy balance leading to malnutrition in these patients is more likely due to elevated energy expenditure, rather than decreased energy intake. This implies that monitoring total caloric intake may not be sufficient in identifying patients at risk of MCI or DE. Moreover, monitoring REE thorough indirect calorimetry, as performed in previous studies, may not be feasible in a large-scale population. Therefore, monitoring %E P:C could serve as a more practical, reliable tool to detect changes in food preferences that may indicate a higher risk of MCI or DE.

In this study, we observed a significant association between the protein-to-carbohydrate ratio and cognitive impairment in patients with type 2 diabetes (T2DM). It is known that stable blood glucose levels are essential for maintaining brain function, as the brain relies heavily on a continuous supply of glucose for energy [52]. Higher protein intake has been shown to improve insulin sensitivity and enhance glucose uptake, reducing glycemic variability that negatively affects both cognitive function and diabetes management [53]. Diets with a higher protein content or a balanced protein-to-carbohydrate ratio may be particularly beneficial for older adults with T2DM, as such diets promote metabolic regulation while mitigating the risk of cognitive decline, including mild cognitive impairment (MCI) and dementia, both of which are more common in this population [10]. Therefore, managing the protein-to-carbohydrate ratio may represent a viable dietary strategy to improve both metabolic and cognitive health in individuals with diabetes [54].

Our study has several limitations. First, the sample is small, and it is a cross-sectional study from a single medical center; therefore, the findings presented here should be interpreted with caution. Even though we found that a low %E P:C ratio was associated with lower cognitive function, this does not prove causality. The association between a lower %E P:C and cognitive impairment suggests that cognitive decline itself may contribute to changes in dietary intake, rather than a low %E P:C being solely a cause of cognitive impairment. This could create a feedback loop where cognitive impairment exacerbates dietary inadequacies, further accelerating cognitive decline. The diet was assessed using a food frequency questionnaire, which is susceptible to omissions of food items and may not accurately reflect habitual dietary behavior. However, it remains the most valid and widely used tool for gathering dietary information in observational studies. Other experts argue that the use of food frequency questionnaires is appropriate for ranking individuals based on their food and nutrient intake, as performed in the present study [55,56]. By including patients with cerebrovascular disease, we recognize the potential for cognitive impairment associated with these events. However, we chose not to exclude these patients to enhance the ecological validity of our findings. It is important to consider the impact of cerebrovascular disease on cognitive outcomes when interpreting our results. Future research may benefit from stratifying patients based on the presence or absence of cerebrovascular disease to better understand its effect on cognitive function in T2DM populations.

Prospective multicentric longitudinal studies are needed to fully clarify how low %E P:C contributes to the genesis of MCI and its progression to DE and to assess the possibility that the %E P:C ratio can be a tool to detect DE or MCI.

## 5. Conclusions

We highlight the importance of the protein-to-carbohydrate energy ratio as a risk factor for cognitive impairment in elderly individuals with T2DM. Monitoring and optimizing the %E P:C ratio may provide an early detection tool for cognitive decline. Furthermore, the optimal protein intake suggested in this study exceeds current dietary guidelines, advocating for revised protein recommendations in this high-risk population to better prevent cognitive deterioration.

## Figures and Tables

**Table 1:** Demographic, clinical characteristics, and dietary composition among MOCA groups. Each value is given as median (interquartile range). Trend *p*-values were obtained through linear regression, with MOCA treated as a categorical variable and clinical or dietary variables as continuous. For categorical variables, we applied the chi-square test among groups. BMI, body mass index; GLP-1RA, glucagon-like peptide-1 receptor agonists; iDPP4, dipeptidyl peptidase-4 (DPP-4) inhibitors; iSGLT-2, Sodium-Glucose Cotransporters 2 (SGLT-2) inhibitors.

	All Participants (*n* = 192)	Normal Cognitive Function (*n* = 55)	Mild Cognitive Impairment (*n* = 106)	Dementia (*n* = 31)	*p*-Value
Age (years)	71 (65–75)	66 (63–71)	71 (67–76)	72 (71–77)	0.010
Gender (female)	89 (46.4%)	26 (47.3%)	49 (46.2%)	14 (45.2%)	0.982
BMI (kg/m^2^)	30 (27–34)	30 (26–35)	30 (27–33)	29 (27–33)	0.666
Waist circumference (cm)	108 (98–121)	110 (98–119)	107 (98–118)	108 (100–124)	0.938
Time from diagnosis T2DM					0.767
Less than 5 years	8 (4.2%)	3 (5.5%)	4 (3.8%)	1 (3.2%)	
Between 5 and 10 years	14 (7.3%)	6 (10.9%)	6 (5.7%)	2 (6.5%)	
More than 10 years	170 (88.5%)	46 (83.6%)	96 (90.6%)	28 (90.3%)	
Education level					0.363
Less than 13 years	119 (61.5%)	35 (63.6%)	60 (56.6%)	23 (74.2%)	
Between 13 and 16 years	48 (25.0%)	14 (25.5%)	28 (26.4%)	6 (19.4%)	
More than 16 years	26 (13.5%)	6 (10.9%)	18 (17.0%)	2 (6.5%)	
Comorbidities					
Diabetic retinopathy	17 (8.9%)	4 (7.3%)	11 (10.4%)	2 (6.5%)	0.706
Diabetic nephropathy	33 (17.2%)	4 (7.3%)	21 (19.8%)	8 (25.8%)	0.052
Intermittent claudication	2 (1.0%)	1 (1.8%)	1 (0.9%)	0 (0.0%)	0.720
Cerebrovascular accident	10 (5.2%)	4 (7.3%)	6 (5.7%)	0 (0.0%)	0.329
Polyneuropathy	16 (8.3%)	5 (9.1%)	10 (9.4%)	1 (3.2%)	0.530
Diabetic foot	4 (2.1%)	0 (0.0%)	3 (2.8%)	1 (3.2%)	0.436
Atherosclerosis	173 (90.6%)	51 (92.7%)	93 (87.7%)	30 (96.8%)	0.258
Hypertension	165 (85.9%)	46 (83.6%)	92 (86.8%)	27 (87.1%	0.844
Smoking	12 (6.3%)	5 (9.1%)	7 (6.6%)	0 (0.0%)	0.452
Alcohol	9 (4.7%)	3 (5.5%)	5 (4.7%)	1 (3.2%)	0.895
Anti-diabetic drugs					
Insulin	84 (43.8%)	27 (49.1%)	43 (40.6%)	14 (45.2%)	0.570
Metformin	146 (76.0%)	46 (83.6%)	77 (72.6%)	23 (74.2%)	0.290
GLP-1 RA	86 (44.8%)	25 (44.5%)	46 (43.4%)	15 (48.4%)	0.880
i-SGLT-2	45 (23.2%)	14 (25.7%)	25 (23.8%)	6 (19.2%)	0.800
Secretagogues	47 (24.5%)	10 (18.2%)	27 (25.5%)	10 (32.3%)	0.324
iDPP4	84 (43.8%)	24 (43.6%)	42 (39.6%)	18 (58.1%)	0.191
Pioglitazone	3 (1.6%)	0 (0.0%)	3 (2.8%)	0 (0.0%)	0.290
Dietary parameters					
Vegetables (g/day)	621 (439–792)	627 (484–773)	630 (414–798)	587 (410–842)	0.349
Fruits (g/day)	590 (472–737)	572 (434–701)	616 (481–766)	582 (461–704)	0.872
Legumes (g/day)	64 (43–64)	64 (43–64)	64 (32–64)	64 (43–64)	0.860
Cereals (g/day)	101 (88–142)	96 (86–131)	111 (88–151)	99 (88–142)	0.730
Whole cereals (g/day)	75 (12–81)	55 (2–75)	75 (21–88)	58 (0–82)	0.919
Dairy (g/day)	258 (227–338)	270 (227–345)	256 (230–333)	261 (220–338)	0.880
Meat (g/day)	120 (79–163)	116 (81–167)	127 (78–167)	111 (74–126)	0.119
Olive oil (g/day)	15 (15–17)	15 (15–18)	15 (15–17)	15 (15–17)	0.918
Fish (g/day)	68 (48–97)	74 (50–108)	67 (45–90)	61 (48–86)	0.191
Nuts (g/day)	9 (0–24)	11 (0–21)	9 (2–30)	6 (0–13)	0.030
Sweets (g/day)	4 (0–18)	3 (0–13)	4 (0–18)	3 (0–18)	0.843
Alcoholic drinks (g/day)	4 (0–100)	4 (0–94)	8 (0–100)	0 (0–24)	0.533
Nonalcoholic drinks (g/day)	107 (56–250)	121 (64–243)	107 (57–250)	79 (50–225)	0.469
Eggs (g/day)	29 (14–36)	29 (21–36)	29 (14–36)	21 (14–29)	0.083
Total energy (kcal/day)	2006 (1642–2512)	1966 (1597–2607)	2061 (1731–2605)	1713 (1590–2279)	0.115
Protein (g/day)	95 (77–114)	94 (78–115)	97 (77–117)	88 (76–102)	0.044
Lipids (g/day)	89 (70–112)	91 (71–106)	91 (72–115)	79 (63–91)	0.093
Carbohydrates (g/day)	246 (205–308)	230 (192–305)	250 (207–314)	246 (206–295)	0.873
Fiber (g/day)	41 (34–50)	38 (34–48)	42 (34–51)	43 (31–49)	0.727
% E Protein	19 (17–20)	19 (17–21)	18 (16–20)	19 (17–20)	0.879
% E Lipids	38 (33–43)	40 (34–43)	38 (33–43)	37 (33–41)	0.649
% E Carbohydrates	50 (46–54)	48 (45–53)	50 (46–55)	50 (48–54)	0.006

**Table 2:** Clinical variables and cognitive function among tertiles of protein-to-carbohydrate ratio (% E P:C). Each value is given as median (interquartile range). Trend *p*-values were calculated using linear regression, with the carbohydrate-to-protein ratio treated as a categorical variable and laboratory variables as continuous data. HbA1c, hemoglobin A1c; LDL, Low Density Lipoprotein; HDL, High Density Lipoprotein.

	Tertile 1 (*n* = 64)	Tertile 2 (*n* = 64)	Tertile 3 (*n* = 64)	*p*-Value Trend
	%E P:C < 0.340	%E P:C [0.340–0.415]	%E P:C > 0.415	
Anthropometric and clinical variables				
Age (years)	73 (67–78)	70 (65–75)	69 (64–73)	<0.001
Gender (female)	26 (40.6%)	36 (56.3%)	27 (42.2%)	0.860
BMI (kg/cm^2^)	29 (26–35)	30 (27–33)	30 (27–34)	0.863
Waist circumference (cm)	110 (98–119)	107 (98–118)	108 (100–124)	0.782
Systolic blood pressure (mmHg)	146 (130–158)	140 (129–160)	145 (130–154)	0.742
Diastolic blood pressure (mmHg)	76 (70–84)	80 (71–89)	80 (74–89)	0.051
Hear rate (bpm)	77 (71–84)	80 (72–88)	79 (76–84)	0.618
Glucose (mg/dL)	134 (108–171)	136 (108–174)	135 (111–156)	0.682
HbA1c (%)	7.3 (6.5–8.0)	7.4 (6.4–8.1)	7.2 (6.6–8.2)	0.984
Total cholesterol (mg/dL)	148 (127–171)	158 (132–176)	146 (124–175)	0.925
HDL cholesterol (mg/dL)	41 (35–51)	44 (36–52)	42 (37–48)	0.882
LDL cholesterol (mg/dL)	76 (55–96)	82 (67–98)	76 (56–96)	0.501
Triglicerides (mg/dL)	127 (96–166)	123 (92–171)	149 (102–209)	0.228
Cognitive function				
Self-Administered Gerocognitive Exam (SAGE)	15.0 (12.0–17.0)	16.0 (13.0–18.8)	17.0 (14.3–19.0)	0.005
Normal cognitive function	19 (29.7%)	28 (43.8%)	3453.1%)	0.008
Mild cognitive impairment	18 (28.1%)	14 (21.9%)	1421.9%)	
Severe Cognitive Impairment or dementia	27 (42.2%)	22 (34.4%)	1625.0%)	
Montreal Cognitive Assessment (MOCA)	21.0 (18.0–23.0)	22.0 (19.0–24.0)	22.5 (20.0–25.0)	0.014
Normal cognitive function	13.0 (20.3%)	19.0 (29.7%)	23.0 (35.9%)	0.044
Mild cognitive impairment	38.0 (59.4%)	34.0 (53.1%)	34.0 (53.1%)	
Severe Cognitive Impairment or dementia	13.0 (20.3%)	11.0 (17.2%)	7.0 (10.9%)	

**Table 3:** Association of cognitive function scales (MoCA and SAGE) with protein-to-carbohydrate ratio (by tertiles and for each increase of 0.2 units). Linear regression models were used to evaluate the association between cognitive function (MoCA and SAGE tests) and protein-to-carbohydrate ratio (tertiles and per each decrease of 0.2 units). Results are expressed as beta coefficients (95% CIs). * Models were adjusted for age (years), gender (male/females), diabetes duration (<5 years; 5–10 years; >10 years), education level (<13 years; 13–16 years; >16 years), total energy intake (kcal/day).

			Tertile 1 (*n* = 64)	Tertile 2 (*n* = 64)	Tertile 3 (*n* = 64)	*p*-Value Trend	%E P:C	*p*-Value
			%E P:C < 0.340	%E P:C [0.340–0.415]	%E P:C > 0.415		(Per Each 0.2 Units)	
Montreal Cognitive Assessment (MOCA)								
	Visuospatial/Executive Function							
		Crude Model	0 (reference)	0.141 (−0.197–0.478)	0.109 (−0.228–0.447)	0.523	0.035 (−0.093–0.154)	0.635
		Adjusted Model *	0 (reference)	0.103 (−0.231–0.436)	0.064 (−0.272–0.400)	0.714	0.039 (−0.099–0.166)	0.592
	Naming							
		Crude Model	0 (reference)	0.188 (0.055–0.320)	0.156 (0.024–0.289)	0.022	0.160 (0.011–0.313)	0.027
		Adjusted Model *	0 (reference)	0.223 (0.090–0.356)	0.187 (0.053 –0.322)	0.008	0.201 (0.039–0.355)	0.006
	Attention							
		Crude Model	0 (reference)	0.125 (−0.375–0.625)	0.312 (−0.187–0.812)	0.218	0.112 (−0.002–0.243)	0.123
		Adjusted Model *	0 (reference)	0.150 (−0.328–0.628)	0.323 (−0.159–0.806)	0.186	0.146 (0.016–0.289)	0.046
	Language Fluency							
		Crude Model	0 (reference)	0.203 (−0.143–0.549)	0.156 (−0.190–0.502)	0.374	0.100 (−0.043–0.233)	0.168
		Adjusted Model *	0 (reference)	0.141 (−0.190–0.471)	0.100 (−0.234–0.434)	0.562	0.112 (−0.039–0.273)	0.128
	Abstraction							
		Crude Model	0 (reference)	0.172 (−0.110–0.454)	0.219 (−0.063–0.501)	0.127	0.054 (−0.088–0.215)	0.457
		Adjusted Model *	0 (reference)	0.139 (−0.142–0.420)	0.212 (−0.071–0.496)	0.141	0.068 (−0.096–0.224)	0.353
	Memory							
		Crude Model	0 (reference)	0.406 (−0.094–0.906)	0.625 (0.125–1.125)	0.014	0.152 (0.024–0.289)	0.035
		Adjusted Model *	0 (reference)	0.213 (−0.286–0.713)	0.494 (−0.010–0.998)	0.054	0.133 (0.004–0.274)	0.069
	Orientation							
		Crude Model	0 (reference)	−0.031 (−0.240–0.178)	0.156 (−0.053–0.365)	0.143	0.075 (−0.056–0.197)	0.301
		Adjusted Model *	0 (reference)	−0.028 (−0.240–0.183)	0.192 (−0.022–0.405)	0.074	0.109 (−0.033 –0.237)	0.136
	Total MOCA							
		Crude Model	0 (reference)	1.109 (−0.198–2.417)	1.641 (0.333 –2.948)	0.014	0.171 (0.059–0.297)	0.017
		Adjusted Model *	0 (reference)	0.874 (−0.358–2.107)	1.441 (0.197–2.685)	0.023	0.195 (0.072–0.326)	0.007
Self-Administered Gerocognitive Exam (SAGE)								
	Orientation							
		Crude Model	0 (reference)	0.031 (−0.191–0.254)	0.172 (−0.050–0.394)	0.128	0.133 (0.023–0.242)	0.067
		Adjusted Model *	0 (reference)	0.040 (−0.189–0.269)	0.206 (−0.025–0.437)	0.078	0.161 (0.052–0.270)	0.028
	Naming							
		Crude Model	0 (reference)	0.016 (−0.149–0.180)	−0.016 (−0.180–0.149)	0.851	−0.056 (−0.191–0.091)	0.440
		Adjusted Model *	0 (reference)	0.005 (−0.163–0.172)	−0.019 (−0.188–0.149)	0.817	−0.048 (−0.195–0.098)	0.512
	Similarities							
		Crude Model	0 (reference)	0.219 (−0.122–0.559)	0.406 (0.066 –0.747)	0.019	0.075 (−0.061–0.217)	0.299
		Adjusted Model *	0 (reference)	0.193 (−0.145–0.532)	0.410 (0.068–0.751)	0.019	0.083 (−0.069–0.236)	0.259
	Calculation							
		Crude Model	0 (reference)	0.109 (−0.162–0.381)	0.141 (−0.131–0.412)	0.308	0.096 (−0.041–0.230)	0.186
		Adjusted Model *	0 (reference)	0.093 (−0.185–0.371)	0.124 (−0.157–0.4049	0.386	0.099 (−0.043–0.242)	0.178
	Construction							
		Crude Model	0 (reference)	0.438 (−0.031–0.906)	0.281 (−0.187–0.750)	0.239	0.068 (−0.072–0.208)	0.346
		Adjusted Model *	0 (reference)	0.467 (0.016–0.919)	0.272 (−0.183–0.728)	0.254	0.088 (−0.059–0.232)	0.232
	Verbal Fluency							
		Crude Model	0 (reference)	−0.047 (−0.170–0.077)	−0.001 (−0.124–0.124)	1.000	0.006 (−0.075–0.090)	0.929
		Adjusted Model *	0 (reference)	−0.058 (−0.185–0.069)	−0.009 (−0.137–0.728)	0.905	0.010 (−0.056–0.084)	0.896
	Executive Function							
		Crude Model	0 (reference)	0.313 (−0.043 –0.668)	0.438 (0.082–0.793)	0.016	0.180 (0.058–0.309)	0.013
		Adjusted Model *	0 (reference)	0.316 (−0.041–0.673)	0.494 (0.134–0.854)	0.008	0.209 (0.081–0.327)	0.004
	Memory							
		Crude Model	0 (reference)	0.172 (−0.117 –0.460)	0.281 (−0.007–0.570)	0.055	0.089 (−0.040–0.229)	0.219
		Adjusted Model *	0 (reference)	0.146 (−0.123–0.416)	0.253 (−0.019–0.525)	0.068	0.103 (−0.054–0.252)	0.159
	Total SAGE							
		Crude Model	0 (reference)	1.250 (0.065 –2.435)	1.703 (0.518–2.888)	0.005	0.162 (0.035–0.303)	0.025
		Adjusted Model *	0 (reference)	1.202 (0.145 –2.259)	1.731 (0.664–2.797)	0.002	0.212 (0.068–0.352)	0.004

**Table 4:** Crude and adjusted odds ratio (OR) of protein-to-carbohydrate ratio (as dichotomic variable using optimal cutoff value and per each 0.2 units) associated with MCI or dementia (vs. normal cognitive function as reference).

	Crude OR	(95% CI for OR)	*p*-Value	Adjusted OR *	(95% CI for OR)	*p*-Value
Protein-to-carbohydrate ratio < 0.375 units	1.996	(1.049–3.800)	0.035	2.089	(1.019–4.283)	0.044
Protein-to-carbohydrate ratio (per each decrease of 0.2 units)	2.413	(1.155–5.043)	0.019	2.544	(1.121–5.775)	0.026

* Adjusted for age (years), gender (male/female), duration of diabetes (<5 years/5–10 years/>10 years), education level (<13 years/13–16 years/>16 years), total energy intake (kcal/day).

## Data Availability

The original contributions presented in the study are included in the article, further inquiries can be directed to the corresponding author. The data are not publicly available due to privacy reasons.
